# P-1037. Hemodialysis-Related Interventions for Reducing Central Line-Associated Bloodstream Infections at a Veterans Administration Hospital

**DOI:** 10.1093/ofid/ofaf695.1232

**Published:** 2026-01-11

**Authors:** Jose Antonio Mansen Arrieta, Aderonke Badejogbin, Joseph Roshni, Annabelle Tipan, Boult Zanzine, Eduardo Herrera, Sheeba Mon Mathew, Sherry R Reid, Ikwo K Oboho

**Affiliations:** UT Southwestern Medical Center, Dallas, TX; Dallas Veteran Affairs, Dallas, Texas; Dallas Veteran Affairs, Dallas, Texas; Dallas Veteran Affairs, Dallas, Texas; Dallas Veteran Affairs, Dallas, Texas; Dallas Veteran Affairs, Dallas, Texas; Dallas Veteran Affairs, Dallas, Texas; Veterans Affairs North Texas Health Care System, Dallas, Texas; VA North Texas Healthcare System, UT Southwestern Medical Center Dallas, TX, Dallas, TX

## Abstract

**Background:**

Central Line-Associated Bloodstream Infection (CLABSI) in Dialysis Patients Significantly Increases Morbidity and is the Second Leading Cause of Death in this Group. According to the Centers for Disease Control and Prevention, Adults on Hemodialysis (HD) are more likely to Develop a Bloodstream Infection compared to Non-HD Patients.

**Figure:**
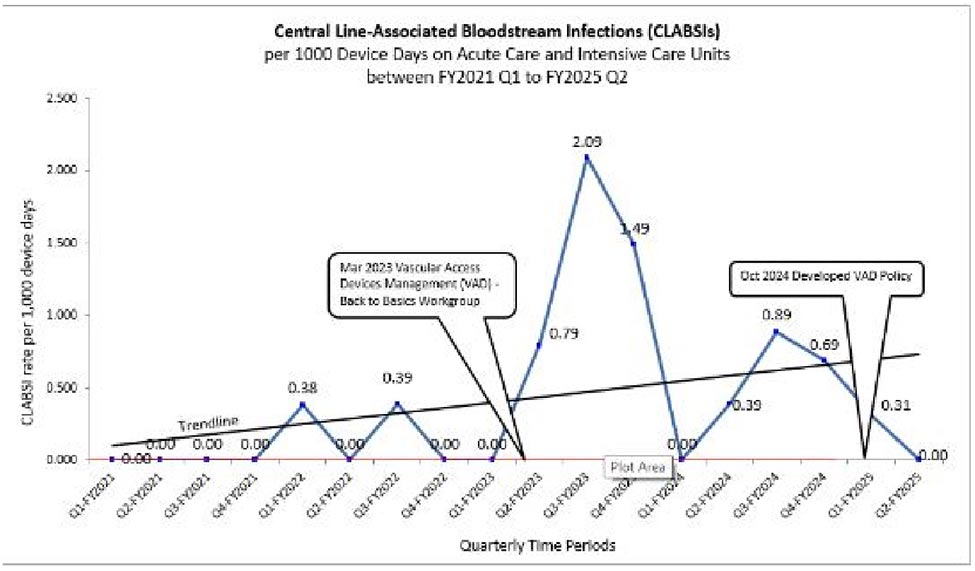
Central line-associated bloodstream infection (CLABSI) rates, Veteran Affairs North Texas Health Care System, Fiscal Year (FY)2021 to FY2025 Quarter 2

**Methods:**

From January – March 2023, Rising CLABSI Rates among HD Patients at the Veterans Administration North Texas Heath Care System prompted Infection Prevention and Control (IPC) to Intensify Observations in the Acute and Critical Care Units. This Revealed Inconsistent Care and Maintenance Practices for Vascular Access Devices (VAD) and the Absence of a Facility VAD policy. Central venous catheter Dressings were Changed every Clinic Visit or every 3 days, instead of the Recommended 7 days. Nurses lacked a Standardized Process and because Dressing Kits were Incomplete, Nurses often Collected Supplies mid-procedure, Increasing the Risk for Catheter Contamination. There was Inconsistent use of Chlorohexidine Gluconate (CHG), CHG-impregnated foam discs, and the disinfection of catheter hubs with alcohol after every use.

In August 2024, IPC led an interdisciplinary team including the HD and vascular access teams to develop a VAD policy and implement a new HD dressing change kit. Nurses were trained in aseptic non-touch technique. The new kit included personalized step-by-step visual guidance, hand sanitizer, sterile gloves, skin protectant, oil-based skin adhesive, alcohol wipes, CHG- Impregnated Foam Disc, and a Transparent Dressing. Alcohol-based CHG solution was used as the Primary Antiseptic prior to Dressing Placement. Scheduled 7-day Dressing changes were Reinforced.

**Results:**

CLABSI rates in the Acute and Critical Care Units decreased from 9 per 1000 Device Days for Fiscal Year (FY) 2023 (Oct 2022 – Sep 2023) to 5 per 1000 Device Days for FY24 (Oct 2023 – Sep 2024). For FY25 Q2 (Oct 2024 – March 2025), the CLABSI rate declined to 0.15 per 1000 device days.

HD-related CLABSI decreased by 58%: 7 of 9 Cases (77.8%) for FY23 to 1 of 5 Cases (20%) for FY24.

**Conclusion:**

HD-related CLABSI Declined by over 50% within a year of Implementation of the New Standardized HD Dressing Change Kit and Improved VAD Maintenance Procedures.

**Disclosures:**

All Authors: No reported disclosures

